# NVVC/NHJ Durrer prizes 2018

**DOI:** 10.1007/s12471-019-1278-6

**Published:** 2019-04-10

**Authors:** J. J. Piek

**Affiliations:** AMC Heart Center, Amsterdam UMC, location AMC , Amsterdam, The Netherlands

The NVVC/NHJ publication prizes are named after one of the founding fathers of Dutch Cardiology, professor D. Durrer (1918–1984) who was the chair of the department of Cardiology at the former Wilhelmina Gasthuis in Amsterdam. He was an outstanding pioneer in the field of electrophysiology with classical works on the electric activation of the heart in the 1960s and 1970s [1]. In 1972, he also founded the Interuniversity Cardiology Institute of the Netherlands (ICIN). Moreover, the Durrer Center, founded in 2008, carries his name in his memory. The Durrer Center is a national multidisciplinary collaboration of academic research institutes in the field of cardiology, genetics and biostatistics.

The Netherlands Heart Journal, the official journal of the Netherlands Society of Cardiology (NVVC), awarded the Durrer prizes to two outstanding NHJ articles published in 2018. These articles are selected based upon their originality and scientific quality as well as the number of citations. The two articles were selected out of a total of 123 articles published in the NHJ 2018.

The first article entitled ‘Extracorporeal life support in cardiogenic shock: indications and management in current practice’ is a review of veno-arterial extracorporeal life support (VA-ECLS). This device is increasingly being used as a cardiac and circulatory support modality with the ability to provide immediate stabilisation in patients with otherwise refractory cardiogenic shock [2]. This review was carried out to assess the current available evidence in the absence of randomised trials. Observational studies on VA-ECLS have suggested a reduction in mortality as compared to conventional treatment. Management of VA-ECLS is complex and requires constant support of trained personnel. This review describes the indications, daily clinical management and complications of VA-ECLS in patients with cardiogenic shock refractory to conventional strategies.

The other selected article is entitled ‘Nationwide claims data validated for quality assessments in acute myocardial infarction in the Netherlands’ [3]. In the Netherlands all reimbursements of the Dutch hospitals are processed by insurance companies and registered by a national diagnosis coding system. The data of patients with ST-elevation myocardial infarction and non-ST-elevation myocardial infarction were retrieved from the national coding system, including the prescription of medication during the first year of follow-up. The data from the national diagnosis coding system was compared with the local databases from four selected hospitals. The data retrieved from the coding systems were compared with the local databases to validate diagnosis and treatment coding, to validate the hospital where follow-up had taken place and to validate the follow-up medical treatment after 365 days. The results of this study show that nationwide routinely collected claims data in patients with acute coronary syndromes are highly accurate. These findings offer the opportunity to use national claims data for quality assessments of cardiac care.

The first authors of these articles received an educational grant, provided by the NVVC, at the Annual Spring Meeting of the NVVC held at the Postillion Convention Centre WTC in Rotterdam, the Netherlands, on 11 and 12 April 2019. The NHJ would like to congratulate the authors with these awards and thank them for submitting their excellent work to our journal. The editorial board of the NHJ hopes that the Durrer prizes act as a stimulus for authors to send their best paper to our journal.
